# Hunting of mammals by central chimpanzees (*Pan troglodytes troglodytes*) in the Loango National Park, Gabon

**DOI:** 10.1007/s10329-020-00885-4

**Published:** 2021-01-08

**Authors:** Harmonie Klein, Gaëlle Bocksberger, Pauline Baas, Sarah Bunel, Erwan Théleste, Simone Pika, Tobias Deschner

**Affiliations:** 1 Department of Primatology, Max Planck Institute for Evolutionary Anthropology, Deutscher Platz 6, 04103 Leipzig, Germany; 2 Comparative BioCognition, Institute of Cognitive Science, University of Osnabrück, Artilleriestrasse 34, 49076 Osnabrück, Germany

**Keywords:** Chimpanzee, Hunting behaviour, Mammals, Loango, Nutrient surplus hypothesis

## Abstract

The predation and consumption of animals are common behaviours in chimpanzees across tropical Africa. To date, however, relatively little is known concerning the hunting behaviour of central chimpanzees (*Pan troglodytes troglodytes*). Here, we provide the first direct observations of hunting behaviour by individuals of the newly habituated Rekambo community in the Loango National Park, Gabon. Over a period of 23 months (May 2017 to March 2019), we observed a total of 61 predation attempts on eight mammal species, including four monkey species. The two most frequently hunted species were two monkey species (*Cercocebus torquatus,* *Cercopithecus nictitans*), which are not hunted at other long-term field sites. The majority of predation events observed involved parties of an average of eight individuals, mainly adult males, with hunting success being higher with increasing numbers of participants. Hunting occurred all year round, but hunting rates increased in the dry season, the period of high fruit availability in the Loango National Park. These results are in line with the nutrient surplus hypothesis which explains seasonal variation in hunting behaviour in several populations of eastern chimpanzees (*Pan troglodytes schweinfurthii*: e.g., Mahale, Tanzania; Ngogo, Uganda). Finally, with a hunting frequency of 2.65 hunts per month, the Rekambo community had higher hunting rates than other sites (Bossou, Republic of Guinea; Kahuzi-Biega, Democratic Republic of Congo; Budongo, Uganda) where red colobus monkeys are also absent. We discuss these results and compare them to patterns at other long-term sites.

## Introduction

Hunting has been suggested as a milestone in the evolutionary trajectory of human sociality and life history traits (Kaplan et al. 2005). Meat is a crucial energy resource and is consumed at higher rates than in any other primate species (Butynski 1982; Hohmann 2009; Pereira and Vicente 2013). Hence, it has been suggested that the onset of hunting marks a shift in the diets of our last common ancestors (Wood and Gilby 2019). A better understanding of the hunting abilities of one of humankind’s closest living relatives, the chimpanzee (*Pan troglodytes*), may therefore shed crucial light on the evolutionary precursors of hominid hunting behaviours. It has been shown that chimpanzees predate and consume a diverse set of animal species, ranging from arthropods (e.g., termites: *Macrotermes *spp.; ants: *Dorylus *spp.), reptiles (e.g., tortoises, *Kinixys erosa*) and birds (e.g., guineafowl, *Guttera pucherani*; coucal, *Centropus leucogaster*) to mammals (e.g., monkey: *Colobus spp.*, *Cercopithecus spp*.; ungulates: *Cephalophus spp*.) (Wrangham 1977; Goodall 1986; Watts and Mitani 2002; Yu et al. 2013; Pika et al. 2019). Concerning mammals, predation has been observed at all long-term study sites including Gombe (Goodall 1986) and Mahale (Nishida et al. 1979) in Tanzania, Ngogo (Mitani and Watts 2001), Budongo (Newton-Fisher 2007) and Kanyawara (Gilby and Wrangham 2007) in Uganda, Taï in Côte d’Ivoire (Boesch and Boesch 1989), Bossou in Guinea (Sugiyama and Koman 1987), Fongoli in Senegal (Pruetz 2006), and Goualougo in the Republic of Congo (Morgan and Sanz 2006). Non-human primates, and especially red colobus monkeys (*Piliocolobus *spp.), are the preferred prey species across sites (Wrangham and van Zinnicq Bergmann Riss 1990; Uehara et al. 1992; Stanford et al. 1994; Mitani and Watts 1999; Hosaka et al. 2001; Boesch 2002; Gilby et al. 2017), followed by ungulates (e.g., Goodall 1986) and rodents (e.g., Alp 1993). Communities differ with regard to hunting frequencies and techniques (Teleki 1973; Uehara et al. 1992). For instance, chimpanzees at Taï and Ngogo show the highest observed frequencies (4.5 and 4 times/month, respectively; Boesch and Boesch 1989; Mitani and Watts 1999). At Mahale, the hunting frequency initially was relatively low (1.6 times/month; Takahata et al. 1984). However, Hosaka and colleagues (2020) note that the hunting activity of the chimpanzees at Mahale have exhibited a significant increase over the past 50 years, becoming comparable to the rate at Gombe (2.6 times/month; Goodall 1986).

Concerning hunting frequencies, scholars have suggested that frequencies may be influenced by food availability and have proposed two hypotheses: the nutrient shortfall hypothesis (Teleki 1973) and the nutrient surplus hypothesis (Gilby et al. 2006). The nutrient shortfall hypothesis postulates that hunting frequency increases to compensate for reduced fruit availability. In contrast, the nutrient surplus hypothesis proposes that hunting occurs more often at times when energy reserves are high and chimpanzees have enough energy to invest in a costly food acquisition strategy such as hunting. To date, results from several long-term study sites are mixed. Some communities engage in higher frequencies of hunting in periods of food shortage (e.g., Gombe: Wrangham 1977; Pusey et al. 2005), some between low and high fruit abundance (Taï: Boesch and Boesch-Achermann 2000), and others in periods of high fruit abundance (e.g., Mahale: Uehara 1997; Ngogo: Watts and Mitani 2002). Here, we investigated which of these two hypotheses best explained the patterns of hunting behaviour observed in a population of central chimpanzees (*Pan troglodytes troglodytes*) living in the Loango National Park in Gabon. This subspecies has been less well studied than other chimpanzee subspecies. In particular, existing studies on hunting behaviour are based on a few direct observations of predation events involving a limited number of prey species, as well as remaining animal tissue in faeces (Makokou: Hladik 1973; Ndoki forest: Kuroda et al. 1996; Goualougo: Morgan and Sanz 2006; Lopé: Tutin and Fernandez 1993; Moukalaba-Doudou: Wilfried and Yamagiwa 2014).

Here, we specifically focused on the following five questions: (1) What are the most preyed upon species by chimpanzees at Loango? (2) How frequently do chimpanzees hunt? (3) Which hunting strategies are employed? (4) What is the hunting success? (5) How is food availability associated with hunting frequency?

## Material and methods

### Study site

The study was conducted in the Loango National Park, Gabon (2°04′S and 9°33′E). Details of the ecological conditions at this field site have been described in Head et al. (2011). The climate fluctuates between a long dry season (from May to September) and a long rainy season (from October to April), with a short dry season in December (Head et al. 2011). In 2017–2018, the mean annual rainfall amounted to 2099 mm and mean annual temperatures ranged from 22.7 °C to 27.8 °C.

In addition to central chimpanzees and lowland gorillas (*Gorilla gorilla gorilla*), nine other primate species live in the Loango National Park (Laurance et al. 2006; Christy et al. 2016). So far, three of them, i.e., grey-cheeked mangabeys (*Lophocebus albigena albigena*, Tutin and Fernandez 1993; Mitani and Watts 1999), moustached monkeys (*Cercopithecus cephus cephodes*, Kuroda et al. 1996) and crowned monkeys (*Cercopithecus pogonias nigripes*, Kuroda et al. 1996), and closely related bushbaby species (*Galago *sp., Hladik 1973; Baldwin 1979; Pruetz 2006; O'Malley 2010; Hosaka et al. 2020) have been reported to be hunted by chimpanzees at other long-term field sites. In addition, other non-primate mammals known to be consumed by chimpanzees at other sites, such as blue duikers (*Cephalophus monticola defriesi*, Hosaka 2015; Ramirez-Amaya et al. 2015; Hobaiter et al. 2017), bay duikers (*Cephalophus dorsalis castaneus*; Morgan et al. 2013), red river hogs (*Potamochoerus porcus*, corresponding to the closely related bushpig—*Potamochoerus larvatus*—in East Africa; Goodall 1986; Mitani and Watts 1999; Hosaka et al. 2020), genets (e.g., *Genetta servalina*) and mongooses (e.g., *Atilax paludinosus pluto*; Nishida et al. 1979; Bogart et al. 2008), also live in the Loango National Park.

### The Rekambo Chimpanzee community

The habituation of the Rekambo Community started in 2005, with the majority of individuals finally habituated to human presence in 2017. During the study period, the community size was estimated to be around 50 individuals, including eight adult males, four adolescent males, approximately 18 adult females, four adolescent females, juveniles and infants. Dominance rank of the adult males of the group was estimated based on unidirectional submissive greetings, “pant-grunts”, between males collected ad libitum (Altmann 1974) during the study period (Bygott 1979).

### Collection of data

Since the beginning of the project in 2005, predation events were opportunistically collected on an ad libitum basis (Altmann 1974). For the present study, we analysed data collected from May 2017 to March 2019, the time period when all adult males were habituated to a level allowing detailed behavioural observations. During the study period, at least one party of chimpanzees was followed on 95% of the days. The data set consists of direct behavioural observations of chimpanzees during a total of 662 days and approximately 7370.6 observation hours. Data collection was performed using customized CyberTracker software (Cyber Tracker 3.441) on water-resistant smart phones (Samsung Galaxy Xcover 3 and Cyrus CS24), and video cameras (Sony Digital 4K video camera) whenever possible. Observed predation events were later transformed into written reports. For the data analyses, we extracted systematic data from each report following parameters used in previous studies on chimpanzee hunting (Boesch and Boesch 1989; Uehara 1997). Particular attention was paid to the following aspects: (1) species predated, (2) hunting strategy, (3) party size, i.e., all chimpanzees visible during the entire hunting event (Boesch 1996) and (4) hunting success. At the time of the study, no data had been collected on tree height at places where hunting occurred. However, a previous study reported an average tree height of 20 m (*N* = 19 tree species; Oelze et al. 2014).

### Definitions of hunting and hunting roles

Following the definition of Packer and Ruttan (1988), we define a hunt as the active pursuit of prey. We considered the start of a hunt from the moment an individual showed interest in a particular prey species or location, i.e., any change in behaviour after the detection of a potential prey species, such as pursuing the prey either on the ground or in the canopy (Boesch and Boesch 1989; Mitani and Watts 1999). A predation event can include multiple killings. The term “successful predation” corresponds to an event where the capture and/or meat consumption was observed (Watts and Mitani 2002). In contrast, “unsuccessful predation” events refer to events where chimpanzees were observed to chase potential prey without catching them. We do not report information about all observed encounters with potential prey species (Busse 1978; Boesch and Boesch 1989). Solitary hunts refer to events where only one individual, within the party, engaged in hunting (Boesch and Boesch 1989).

To describe the hunting strategies involved, i.e., how the chase was carried out and the action of each individual during the hunt, we paid particular attention to the following roles. (a) *Initiator of the hunt:* This term refers to the first individual involved in trying to catch an individual of a given prey species. If a hunt mainly occurred on the ground, the individual that discovered the prey and made the first observable pursuing movement in the direction of the prey (e.g., after hearing vocalizations or noise made by a potential prey’s movement, the individual stops and listens, subsequently runs in the direction of the noise, and begins to walk slowly and silently or watches the canopy) was coded as the initiator. If a hunt mainly took place in the canopy, the first individual who was observed climbing up a tree in the direction of the prey was coded as the initiator (Samuni et al. 2018). (b) *Hunter*: This term refers to an individual that showed via physical action (such as climbing up a tree or moving towards the prey) its motivation to actively participate in the hunt, either in the canopy or on the ground (Boesch and Boesch 1989). (c) *Catcher:* This term refers to the individual who was observed catching the prey. (d) *The first individual seen holding the prey*: This term refers to cases in which a chimpanzee had control of an entire, or almost entire, carcass but the capture was not observed (Goodall 1986).

### Fruit availability measures

Phenology circuits were established in the territory of the Rekambo community in 2006 (Head et al. 2011), and phenology data has been collected since then on a monthly basis. Here, we used the data collected from April 2017 to March 2019, and focused on those 13 species that were represented with more than eight individual trees in the phenology circuit: *Beilschmiedia* sp., *Dacryodes normandii* (Aubrév. & Pellegr.), *Dialium dinklagei* (Harms), *Diospyros dendo* (Hiern), *Duguetia staudtii* (Engl. & Diels; Chatrou), *Hexalobus crispiflorus* (A. Rich.), *Irvingia gabonensis* (Aubry-LeCompte ex O’Rorke Baill.), *Manilkara obovata* (Sabine & G. Don; J.H. Hemsl.), *Pycnanthus angolensis* (Welv.; Warb.), *Sacoglottis gabonensis* (Baill.; Urb), *Staudtia kamerunensis* var. *gabonensis* (Warb.; Fouilloy), *Tieghemella africana* (Pierre) and *Vitex doniana* (Sweet). In addition, we used habitat survey data collected by Head and colleagues (2011) to calculate the density (stem per hectare) and the mean diameter at observers’ mean breast height of each species in the phenology. We calculated the fruit availability index in the same way as Head and colleagues (2011):

$${\mathrm{FAI}}_{m}=\sum_{k=1}^{n}{D}_{k}{B}_{k}{P}_{k},$$

where FAI_*m*_ is the fruit availability index per month, *D*_*k*_ is the density of the species *k*, *B*_*k*_ is the species *k* mean diameter at observers’ mean breast height, and *P*_*k*_ is the percentage of individuals of the species *k* bearing ripe fruits in the month *m*.

## Results

**Fig. 1:**
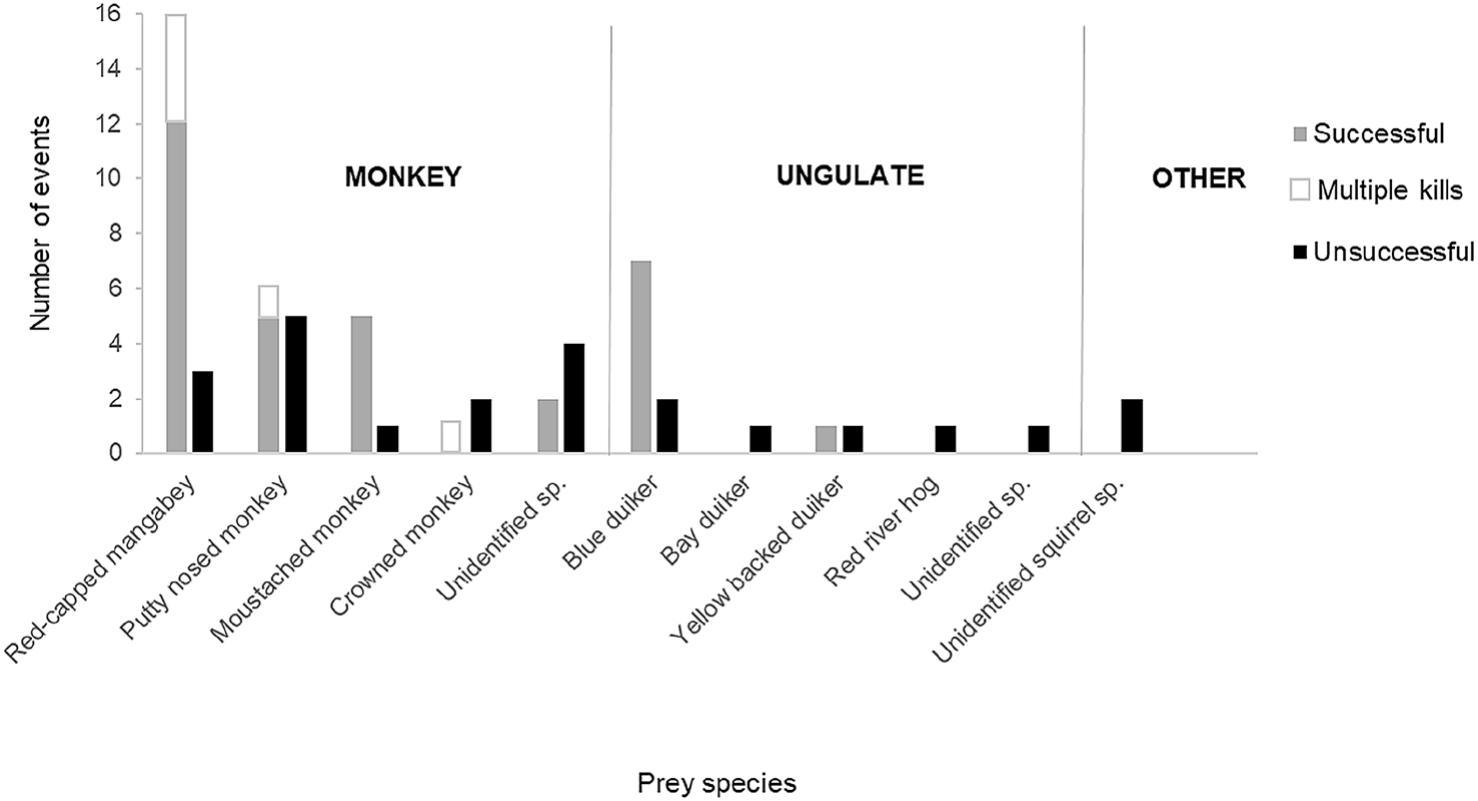
Frequency of predation events on different mammal species by chimpanzees of the Rekambo community in the Loango National Park, Gabon

**Fig. 2:**
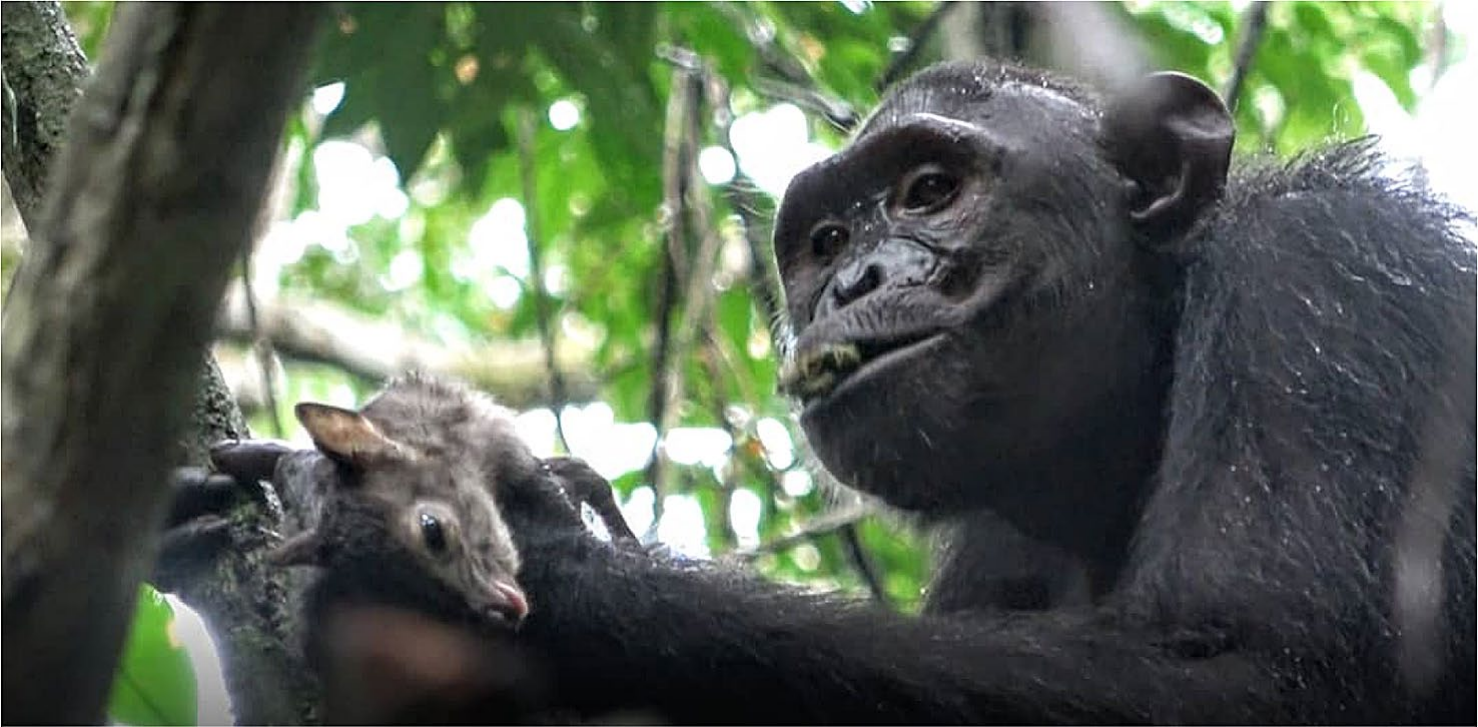
An adolescent chimpanzee male, Arnold, eating a blue duiker. Photo credit: Lara Southern

Overall, a total of 61 predation events, including 38 successful and 23 unsuccessful ones, were observed during the period from May 2017 to March 2019 (Fig. 1). The chimpanzees of the Rekambo community preyed primarily on two monkey and one duiker species (Fig. 2). They hunted at an average frequency of 2.65 hunts per month. Mean party size was eight individuals (ranging from two to 20 individuals), with the mean number of hunters being three individuals (ranging from one to 11 individuals) and involving mainly adult males. Predation events were largely the result of group hunting (*N* = 52); i.e., the hunt involved more than one hunter, and hunters acted simultaneously towards the same prey or group of prey (Boesch and Boesch 1989). We furthermore observed nine solitary hunting attempts, which were however not successful. Linking the data to fruit availability measures showed that predation events occurred more frequently in periods of high fruit availability.

Five predation events involved multiple killings of monkeys, resulting in successful catches of 44 prey items during the 38 successful predation events (Fig. 1). In 12 cases, we were able to observe the entire hunting event starting with individuals approaching the prey, catching the prey and consuming the meat. In 19 cases, we observed the whole predation event starting with individuals approaching the prey but failing to catch it. In 15 cases, the observers lost the chimpanzees during the pursuit and found them again after some minutes holding the prey. In five cases, individuals were found feeding on or carrying a prey and/or prey item. For 10 events the description of the hunt involved only data regarding the species predated and the success of the event.

### Prey species

**Fig. 3:**
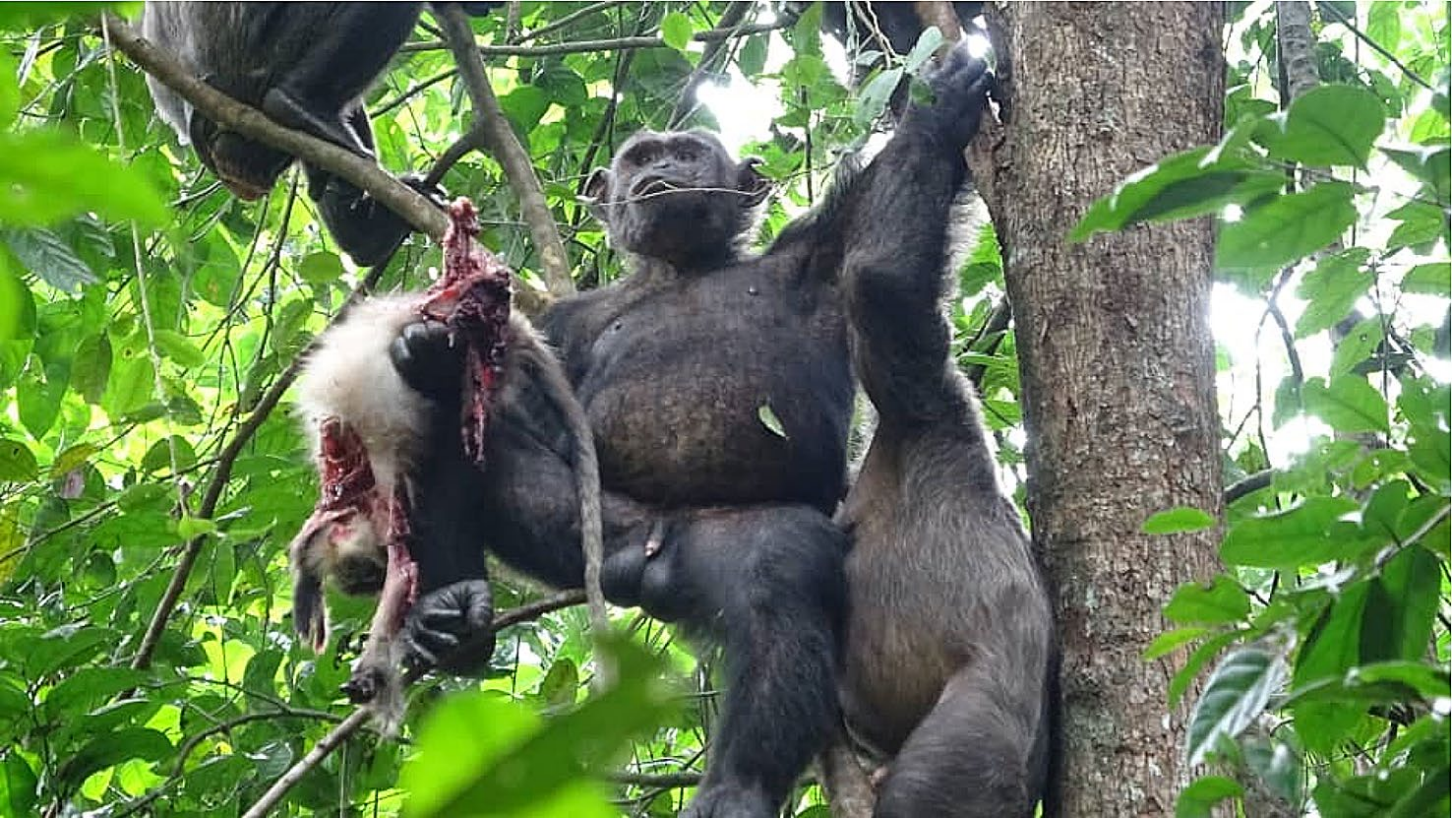
Pandi, an adult chimpanzee male, eating a red-capped mangabey. Photo credit: Alessandra Mascaro

The chimpanzees hunted eight different mammal species (Fig. 1). The most frequent prey species were red-capped mangabeys (*Cercocebus torquatus*; Fig. 3) and putty-nosed monkeys (*Cercopithecus nictitans*). Concerning ungulates, the chimpanzees mainly hunted blue duikers but were seen chasing a yellow-backed duiker (*Cephalophus silvicultor*) twice, killing and consuming it in one of the incidents. They also attempted to hunt a bay duiker, as well as a red river hog. Moreover, on two occasions, solitary chimpanzees were observed trying unsuccessfully to catch a squirrel of an unknown species.

The age class of the prey was rarely documented in the reports (*N* = 22/67). However, 12 preyed upon monkeys (seven red-capped mangabeys, three crowned monkeys and one moustached and putty-nosed monkey), three blue duikers and two yellow-backed duikers were identified as immature individuals. In addition, the hunting attempt on the red river hogs involved an immature individual. Adult prey individuals were only reported in four cases (two red-capped mangabeys, one crowned monkey and one unidentified monkey species).

### Hunting strategies and success

In the majority of observed events, the chimpanzees employed a distinctive hunting sequence: When a potential prey was detected, they became silent and walked quickly in the direction of the prey. One individual, the initiator, regularly took the pole position of the party. One or two chimpanzees would then climb up the tree where the prey had been spotted. During this time, the other individuals would sit silently on the ground, waiting and observing the movements of the individuals in the tree. Frequently, just before the hunters would start to chase the prey, one of them would give a brief but loud vocalization, described by Boesch (1994) and Mitani and Watts (1999). At that moment, some of the individuals on the ground would run under the tree where the prey was located and join the chase. While some additional individuals would climb up the tree, others would stay on the ground. We also observed that some individuals of the party would not take part in any of the hunting-related activities.

On several occasions, we observed a party of chimpanzees actively searching for prey: the chimpanzees would move very silently in single file, similar to the way they move when patrolling, while regularly stopping and gazing into the canopy, scanning and being attentive to arboreal movement. This type of behaviour could be observed several times within a day and always resulted in a hunt if prey and especially monkeys were discovered. On other occasions, the chimpanzees started to hunt after hearing vocalizations of monkeys. Furthermore, instantaneous hunting was also sometimes observed: the chimpanzees would suddenly jump on an approaching prey. Instantaneous hunting was mainly observed with ungulate species (all events) and with squirrels (*N* = 2).

Regarding the preys’ reactions, we observed three attacks from red-capped mangabeys but not from other species. On two of these occasions a female pursued hunters, and on the third one, several individuals were seen charging the chimpanzees.

The analysis of 16 predation events for which the event was witnessed from the beginning to the end showed that the mean duration of a hunt was 22 min (ranging from 1 to 80 min; median: 5 min). In the majority of these cases (*N* = 9), hunting lasted less than 7 min, four lasted between 7 and 30 min, and three lasted more than 30 min.

**Fig.4:**
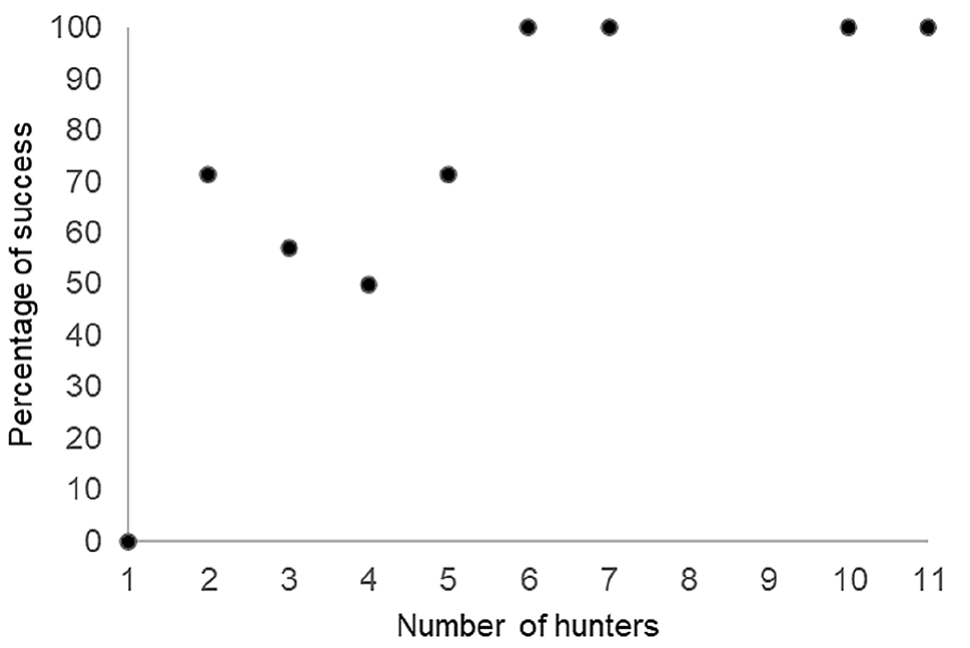
Hunting success in relation to number of hunters

During the 61 predation events, hunting success depended on the number of hunters, with zero success for solitary hunters and increasing success with an increasing number of hunters (Fig. 4). Group hunting occurred most often when there were two to five hunters (42% of the predation events), but the number of hunters could be as many as 11 individuals participating in a single hunt. Party size varied across predation events, and hunting tended to occur more often when chimpanzee party size ranged from three to five individuals (44% of the predation events). However, general party size observed during daily follows tended to be higher, with party sizes ranging from five to ten individuals most often observed (50% of daily follows).

Hunts were most often initiated by Pandi, the alpha male of the Rekambo group (Table 1). He was also the most frequent catcher. In addition, every adult male of the community initiated a hunt at least once. Individuals who had a higher tendency to initiate a hunt were also more frequently observed capturing a prey than non-initiators. Possession was most often observed for the alpha male Pandi as well. An adolescent female was twice observed to join adult males in the initiation of a hunt and was once seen to be the first individual holding the prey. While the alpha male participated in the majority of all hunting events observed, female participation at Loango was observed only twice for an adolescent female and once for an adult female. For 23 events, the participants of the hunt could not be identified because of poor visibility and/or speed of action.

**Table 1:** Hunting participation of members of the Rekambo community

Individuals	Sex	Age class	Dominance rank	Total hours of observation	Number of initiations	Number of captures	First ind. holding prey	Number of times participating
Pandi	Male	Adult	1	4255	7	9	10	24
Chinois	Male	Adult	2	1950	5	1	0	10
Chenge	Male	Adult	3	2710	5	1	4	13
Louis	Male	Adult	4	2350	1	0	3	6
Freddy	Male	Adult	5	3210	0	0	1	15
Onoumbou*	Male	Adult	6	1050	1	0	3	10
Thea	Male	Adult	7	2423	6	2	2	20
Littlegrey	Male	Adult	8	2925	3	1	0	11
Ngonde	Male	Adolescent	9	1590	1	0	4	9
Gump	Male	Adolescent	10	1228	0	0	0	3
Orian	Male	Adolescent	11	2210	3	0	0	12
Arnold	Male	Adolescent	12	1739	0	2	1	3
Joy	Female	Adult	–	1471	0	0	0	1
Gia	Female	Adolescent	–	2868	1	0	1	8

*Individual died during the study period

### Seasonal hunting patterns in relation to fruit availability

**Fig. 5:**
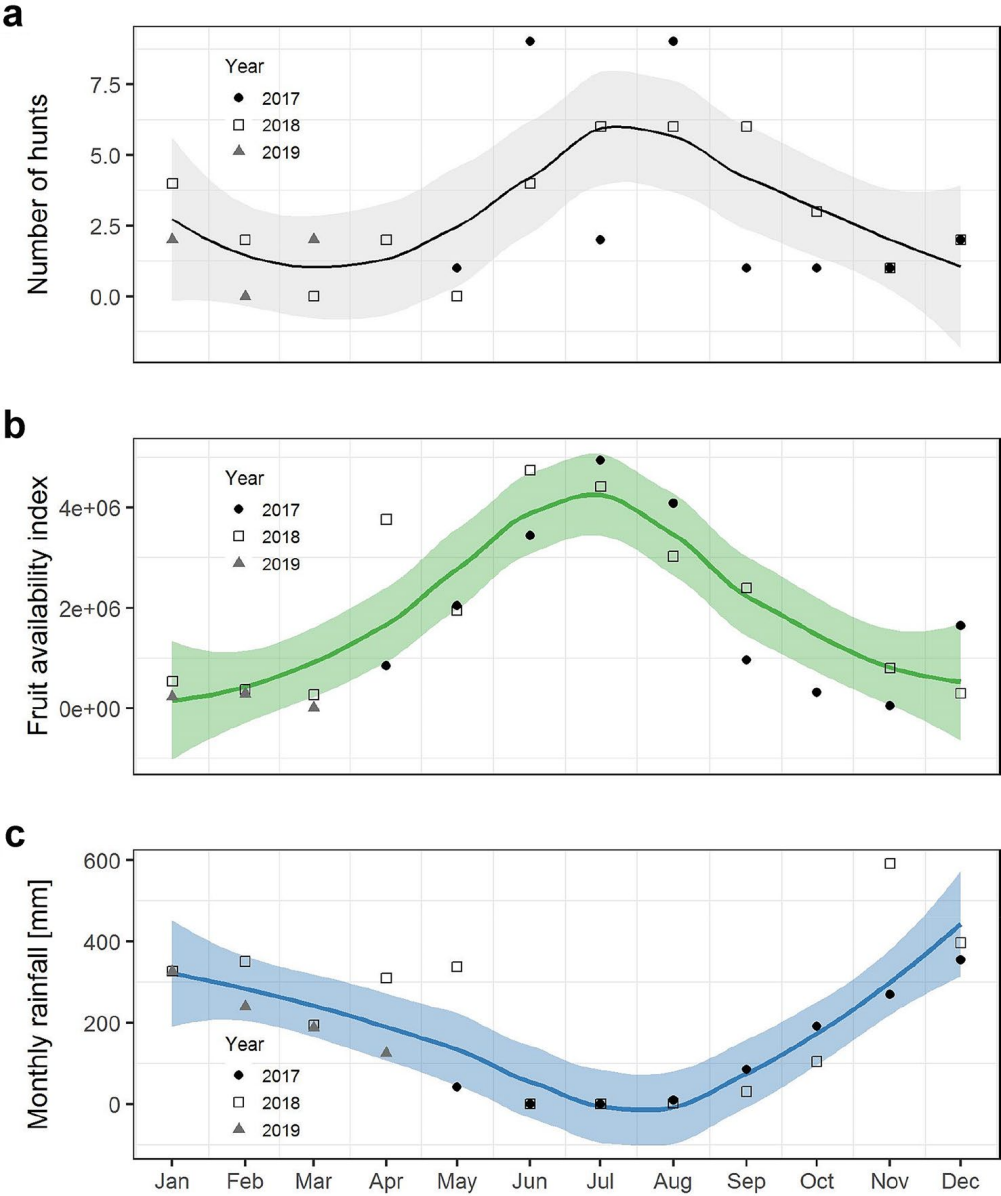
Hunting rate, fruit availability and rainfall. We fitted a smooth function (solid lines) with 95% confidence intervals (shaded area) on (**a**) number of hunts per month from May 2017 to March 2019, (**b**) monthly fruit availability index calculated from April 2017 to March 2019, (**c**) monthly rainfall recorded from May 2017 to March 2019. Symbols represent data for each of the three different years

The Rekambo chimpanzees hunted all year round (Fig. 5). However, predation events were more frequent during the months of June to August 2017 and June to October 2018. These periods correspond to the long dry season and the period of high fruit availability at Loango (Head et al. 2011).

## Discussion

In the present study, we investigated the hunting behaviour of central chimpanzees of the Rekambo community living in the Loango National Park, Gabon, with a special focus on mammal predation and linkage to food availability. We particularly focused on prey species, hunting frequencies, hunting strategy, hunting success and influence of food availability on hunting behaviour.

Overall, our results showed that the Rekambo chimpanzees most frequently hunted two monkey and one duiker species, had intermediate hunting frequencies and were most successful in hunting parties. Predation events consisted of specific behavioural sequences and occurred more often in periods of high fruit availability during dry seasons.

### Prey species

Across the different study sites in Africa, distinct differences exist in relation to the prey species hunted by chimpanzees (Newton-Fisher 2007). The chimpanzees of the Rekambo community hunted mammal species already known to be part of the chimpanzee diet at other sites, but also species never before reported as predated by chimpanzees. For instance, concerning monkeys, at the majority of long-term chimpanzee study sites, the main prey species is the red colobus monkey (Wrangham and van Zinnicq Bergmann Riss 1990; Uehara et al. 1992; Stanford et al. 1994; Mitani and Watts 1999; Boesch 2002). This species is absent at Loango (Laurance et al. 2006; Christy et al. 2016). The main prey species at Loango were the red-capped mangabey and the putty-nosed monkey, two monkey species that have never been reported as prey species at any other long-term field site (Newton-Fisher 2007). There are two possible explanations. First, since these species can only be found from Gabon to the south of Nigeria, with a limited distribution of putty-nosed monkeys found in Côte d’Ivoire and Liberia (Kingdon 1997), the absence of predation of these species at other sites may simply reflect their general absence in the chimpanzee habitat. However, putty-nosed monkeys are present at Lopé (White 1994) and Taï (Boesch and Boesch-Achermann 2000), but predation on them by chimpanzees has never been reported at these sites. Alternatively, the absence of observations on hunting of putty-nosed monkeys may be due to relatively low habituation levels of chimpanzees at Lopé, while at Taï, putty-nosed monkeys exist at very low population densities (Eckardt and Zuberbühler 2004; Bitty and McGraw 2007). Similar to other studies on central chimpanzees at Ndoki forest (*N* = 4/11 predation events reported; Kuroda et al. 1996), the Rekambo chimpanzees hunted and consumed moustached and crowned monkeys.

Concerning ungulate predation, the present study corroborates findings reported for central chimpanzees at Lopé (*N* = 1; blue duiker bones in faeces; Tutin and Fernandez 1993), Ndoki forest (*N* = 1/11 events; unidentified species; Kuroda et al. 1996) and Goualougo (*N* = 1; blue duiker; Sanz 2004). These findings, however, contrast with studies on hunting behaviour in western chimpanzees, where predation on ungulates has only rarely been observed (Fongoli: *N* = 1; bushbuck (*Tragelaphus scriptus*); Pruetz and Marshack 2009; Bossou: *N* = 1; unidentified species; Hirata et al. 2001). Furthermore, in 40 years of observations of three chimpanzee communities living in the Taï National Park, only one case of animal toying (i.e., when chimpanzees catch a prey, manipulate it as to play and do not consume it; e.g., Hirata et al. 2001) on a young blue duiker by juvenile chimpanzees has been reported (Boesch and Boesch 1989). In contrast, predation of ungulate species (e.g., bushbuck, bushpig, several duiker species) is a common behaviour of eastern chimpanzees (e.g., Gombe: Goodall 1986; Mahale: Hosaka and Nishida 2010; Budongo: Hobaiter et al. 2017). The chimpanzees of the Rekambo community hunted four species of ungulates (blue duiker, bay duiker, yellow-backed duiker and red river hog). However, the predation on red river hogs and bay duikers were attempts only, with prey individuals successfully fleeing. Regarding yellow-backed duikers, the first event may qualify as animal toying, where at least two adult males were seen pulling a fawn out of a hole and manipulating it for 10 min before abandoning the injured animal. In the second event, occurring 8 months later, at least three adult individuals killed and consumed a yellow-backed duiker fawn. Hence, this observation provides further evidence for the diversity of chimpanzee feeding ecology (Pika et al. 2019).

When hunting mammals, chimpanzees (Takahata et al. 1984; Goodall 1986; Mitani and Watts 1999) have been reported to focus mainly on immature prey. In the present study, individuals of the Rekambo community attacked six immatures (three blue duikers, two yellow-backed duikers and one piglet) during 14 predation events on ungulates and, in contrast, they caught 12 immatures as well as four adults during the 45 predation events observed on monkeys. The age of the prey individuals, however, could not be assessed for the other events. Hence, the hunting behaviour of the Rekambo chimpanzees seems to show patterns similar to eastern chimpanzees: although they are able to hunt adult individuals, they seem to focus more on immatures. This is perhaps to avoid the aggression of adults (Goodall 1986; Boesch and Boesch 1989), who were seen to attack hunters on three occasions at Loango. Moreover, despite an apparently rather even age class distribution between immatures and adults in putty-nosed monkey and red-capped mangabey groups (Mitani 1989; Arnold and Zuberbühler 2006), the main prey species at Loango, Rekambo chimpanzees seem to hunt immatures more often than adults. However, age of prey, age-sex composition of prey groups and prey reactions at Loango need to be addressed in future studies.

### Hunting frequency

In chimpanzee populations where red colobus monkeys are absent, chimpanzees appear to hunt less frequently or only on occasion (Basabose and Yamagiwa 1997; Yamakoshi 1998; Newton-Fisher et al. 2002). At Loango, however, chimpanzees showed a relatively high rate of hunting, indicating that high hunting rates do not necessitate the presence of red colobus. In comparison to results from other long-term study communities where red colobus monkeys are present, the hunting frequency of the Rekambo chimpanzees was higher than at Kanyawara (Gilby et al. 2017), similar to Gombe and Mahale (Goodall 1986; Hosaka et al. 2020), and lower than at Ngogo (Mitani and Watts 1999) and Taï (Boesch and Boesch 1989). Additionally, in the same data collection period, the Rekambo chimpanzees predated non-mammal vertebrate prey, such as hinge-back tortoises (Pika et al. 2019), at considerably high rates. Therefore, meat consumption by the Rekambo chimpanzees at Loango might be relatively high in comparison to findings from most other long-term study sites. However, further investigations on specific meat consumption rates are needed to assess this aspect in detail.

### Hunting strategies

The distribution of hunting durations at Loango was comparable to other sites. For instance, similar to data from Gombe (Busse 1978) and Mahale (Uehara et al. 1992), the majority of hunting events were relatively short, lasting less than 7 min, with a median of 5 min.

Concerning hunting strategies employed, the general hunting sequence employed and the hunting patrol patterns observed at Loango were similar to the patterns reported for Taï (Boesch and Boesch 1989; Boesch 1994, 2002) and Ngogo (Mitani and Watts 1999): hunting patrols represented a regular pattern in the majority of hunts involving several adult male hunters. Instantaneous hunts were relatively rare at Loango and, in comparison to other sites, more often targeted ungulate species (Goodall 1986; Uehara et al. 1992; Mitani and Watts 1999). Moreover, hunting strategies might vary in relation to prey species targeted. For instance, our preliminary data suggested that chimpanzees are more cautious when approaching mangabeys. This might be due to anti-predation strategies of the different prey species. Indeed, our observations suggested that adult mangabeys seem to sometimes riposte to hunts (with barking, arm waving, short lunges). Moreover, this species is semi-territorial and spends considerable time on the ground (Mitani 1989). Therefore, it is possible that the mangabeys’ capacity to flee on the ground necessitates chimpanzees approaching them more silently to launch a surprise attack.

Individual hunting participation observed at Loango was similar to descriptions from other sites. Some individuals took part more often and/or were more actively engaged in a hunt than others as initiator, hunter or catcher. Following Gilby and colleagues (2015), these individuals potentially qualify as “impact hunters”. Individual variation in participation in hunting activities could not be explained by variation in observation time and habituation level alone. Indeed, all males were fully habituated at the start of the study, and several males less often observed still participated regularly in hunts when present (Table 1). Similarly, rank alone does not seem to explain hunting participation. For instance, while the alpha male participated most frequently in hunts, some lower-ranking individuals, such as Thea, Freddy or Littlegrey, had very high participation rates as well. This result is in contrast to findings from Ngogo, where participation in hunts and hunting success both increased with rank (Watts and Mitani 2002). However, more in-depth investigation in the future is required to confirm this tendency.

Females rarely participated in the hunting action but would readily consume meat, adopting the “wait for someone to capture the prey” tactic (Hosaka et al. 2001). However, because of the low habituation level of females at Loango, it might be premature to make any firm conclusions on this matter. The only case of an adolescent female seen first holding the prey may have been the result of a recovery (Boesch and Boesch 1989), when Gia took a carcass after the catcher dropped it.

### Hunting success

The chimpanzees of the Rekambo community hunted more often in groups than alone, which increased their probability of success. These results are in line with findings from other sites, where hunting success was related to the number of hunters (Boesch 1994) and the party size (Boesch and Boesch 1989; Stanford et al. 1994; Mitani and Watts 1999; Hosaka et al. 2001; Gilby and Wrangham 2007). When hunting monkeys, it appears that at Loango, chimpanzees hunted in relatively large parties, with an average of eight individuals per hunt, while chimpanzees at Taï hunted mainly in parties averaging 3.5 individuals (Boesch et al. 2006). Similar to hunting at Taï, solitary hunts were relatively rare, and when attempted, they were not successful, except when catching duikers. This finding is in contrast to observations at Gombe, where solitarily hunts represent more than 50% of the hunts (Boesch and Boesch 1989).

In addition, group hunting occurred most often when the party size ranged from three to five individuals, while general party size observed during daily follows tended to be higher. This is in line with results from Mahale (Hosaka 2015), where group hunts tended to occur in smaller parties of males. Hosaka suggested that this might be to avoid any costs due to harassment during meat consumption. Interestingly, in our present data set, hunting success reached 100% when the number of hunters was higher than five. Therefore, it may be interesting in future studies to investigate how and why even more hunters regularly engaged in group hunts.

### Influence of food availability on hunting frequency

Gilby and colleagues (2006) tested two hypotheses to investigate how hunting frequencies among chimpanzees may be influenced by food availability. According to the nutrient shortfall hypothesis, we expected to find higher hunting frequencies in the rainy season, which correlated with lower levels of fruit productivity. In contrast, if chimpanzees could afford to invest in costly food acquisition strategies, we expected to find higher hunting frequencies in the dry seasons, which correlate with very high values of fruit at Loango (Head et al. 2011). Our results showed that hunting most frequently occurred in the dry season, therefore providing support for the nutrient surplus hypothesis (Gilby et al. 2006). There were three events where chimpanzees caught a mammal (one red river hog piglet, one yellow-backed duiker fawn and one adult red-capped mangabey) without killing or eating them. All incidents, however, happened during the rainy season. Similar predation and feeding patterns have been observed concerning the consumption of hinge-back tortoises at Loango (Pika et al. 2019). Individuals ignore tortoises from October to November, while they readily consume them between April and September (T.D. and S.P., unpublished data). This finding may suggest that factors other than availability of prey species influence the patterns of hunting seasonality, as described in Mitani and Watts (2005), inspiring future investigation. For instance, ecological factors or, until now, unknown properties of prey species and predators might affect hunting seasonality as well.

In sum, the seasonality patterns of the present study are in line with studies of hunting behaviour at Gombe (Gilby et al. 2006) and Ngogo (Uganda), with chimpanzees investing in hunting more when having surplus energy to engage in this costly activity (Watts and Mitani 2002).

## Conclusion

The hunting behaviour of central chimpanzees showed similarities to and differences from that of eastern chimpanzee populations and patterns of western chimpanzees at Taï concerning the species preyed on, with Loango chimpanzees preying on ungulates and three new species never reported as chimpanzee prey elsewhere. Hunting frequency was also higher than at other sites where chimpanzees are not sympatric to red colobus. The hunting success increased with the number of hunters. These results provide support for the nutrient surplus hypothesis, with chimpanzees investing in hunting mainly at times when fruit availability peaked at Loango National Park. Our results therefore significantly expand our current knowledge of chimpanzee behavioural diversity (Boesch et al. 2020).

## Data Availability

Request for metadata can be submitted to the corresponding author, who is committed to fulfilling all requests.
